# Clinicopathological and molecular features of solid pseudopapillary neoplasms: a retrospective series including a small subset of aggressive cases

**DOI:** 10.3389/pore.2026.1612367

**Published:** 2026-03-09

**Authors:** Erdem Comut, Ahmet Celik, Alper Uguz, Orkan Ergun, Simge Baran, Asuman Argon, Deniz Nart, Funda Yilmaz, Nese Calli Demirkan

**Affiliations:** 1 Department of Pathology, Faculty of Medicine, Pamukkale University, Denizli, Türkiye; 2 Department of Pediatric Surgery, Faculty of Medicine, Ege University, Izmir, Türkiye; 3 Department of General Surgery, Faculty of Medicine, Ege University, Izmir, Türkiye; 4 Department of Pathology, Izmir City Hospital, Izmir, Türkiye; 5 Department of Pathology, Faculty of Medicine, Ege University, Izmir, Türkiye

**Keywords:** clinicopathological features, CTNNB1 mutation, immunohistochemistry, molecular alterations, next-generation sequencing, solid pseudopapillary neoplasm

## Abstract

Solid pseudopapillary neoplasms (SPNs) are rare pancreatic tumors that are indolent but occasionally present with metastatic or locally invasive disease. Although recurrent *CTNNB1* exon 3 mutations define their molecular background, the clinicopathological and molecular features associated with these less common presentations remain incompletely characterized. This retrospective study included 62 patients diagnosed with SPN between 2000 and 2025. Clinicopathological and immunohistochemical features, including β-catenin, progesterone receptor (PR), androgen receptor (AR), and BAP1, were evaluated. Targeted sequencing was performed in a subset of cases with metastatic or locally invasive disease (n = 5). Patients showed a wide age range (8–71 years), female predominance (54/62, 87.1%), and a mean tumor size of 7.2 cm. Lymphovascular invasion was rare (1/59, 1.7%). Metastatic or locally invasive SPNs (n = 8) more frequently showed higher Ki-67 values (median, 5%; range, 1%–15%), increased mitotic activity (2/8, 25%), and capsular/parenchymal invasion (6/8, 75%), while perineural invasion was absent. All tumors demonstrated nuclear β-catenin expression, with PR and AR positivity (50/59, 84.7% and 47/57, 82.5%, respectively). PR expression was higher in AR-positive cases (43/47, 91.5% vs. 6/10, 60%). BAP1 loss was identified in 13/57 cases (22.8%). Targeted sequencing consistently identified *CTNNB1* exon 3 mutations. Additional low-frequency molecular alterations affecting genes involved in cell cycle regulation, chromatin remodeling, and signaling pathways, including *CDKN2A* and *BAP1*, were observed. During a mean follow-up of 97.2 months, distant metastasis occurred in 4/62 patients (6.5%) and locally invasive disease in 4/62 (6.5%), with an overall survival rate of 95%. Overall, these findings highlight the biological heterogeneity of SPNs and indicate that, despite a shared molecular background, aggressive behavior is not defined by a single reproducible pathological or molecular feature.

## Introduction

Solid pseudopapillary neoplasm of the pancreas (SPN), historically known by various names, was formally recognized as a distinct entity in the 2010 World Health Organization (WHO) classification [1, 2]. SPN accounts for approximately 1%–3% of all pancreatic neoplasms [1, 3]. It predominantly affects young women and is not associated with known hormonal or hereditary syndromes, although frequent progesterone receptor (PR) expression suggests a potential role for hormonal signaling in tumor biology [4].

At the molecular level, SPN is defined by constitutive activation of the Wingless/Integrated (Wnt) signaling pathway (Wnt/β-catenin pathway) through exon 3 mutations of the *CTNNB1* gene, leading to nuclear and cytoplasmic accumulation of β-catenin [5–7]. These mutations disrupt the phosphorylation and degradation motifs of β-catenin, most often involving residues D32 or S37, and this disruption results in sustained pathway activation and continuous transcriptional activity of downstream targets [8]. In addition to Wnt/β-catenin signaling, interaction with other pathways, including Hedgehog and androgen receptor (AR) signaling, have been reported [9]. Moreover, loss of BAP1 expression has been reported in a subset of clinically aggressive or metastatic SPNs [10], suggesting that secondary molecular alterations may contribute to biological heterogeneity beyond the canonical Wnt-driven phenotype. Recent multi-omic studies, including integrated methylation and transcriptomic analyses [11], further support that complex regulatory mechanisms may be present in subsets of SPNs extending beyond *CTNNB1* activation.

SPN generally exhibits low-grade malignant potential, with high survival and low recurrence rates after complete resection. Nevertheless, 10%–15% of cases develop metastases, most commonly in the liver or peritoneum, while lymph node involvement remains rare [2, 12]. Although certain histopathological features, such as increased proliferative index and nuclear atypia, have been associated with aggressive behavior, their association with clinical outcome remains variable [12–14]. Because SPNs with aggressive clinical behavior are rare, available clinicopathological and molecular data are limited, and current knowledge is largely derived by retrospective series and case reports [6].

This study aimed to characterize the clinical features of SPNs, to evaluate their histopathological and immunohistochemical findings, and to investigate the molecular features of a subset of cases with metastatic or locally aggressive disease.

## Materials and methods

### Study design and patient selection

A retrospective clinicopathological analysis was performed on 62 patients diagnosed with SPN between 2000 and 2025 in two pathology departments. Clinical variables (age, sex, surgery, tumor site and size, metastasis status) were extracted from electronic medical records, and follow-up data were obtained via outpatient visits, the national electronic patient registry, or direct patient contact.

Clinical aggressiveness was defined as the presence of distant metastasis or invasion into adjacent tissues or organs, reflecting locally aggressive behavior.

### Histopathological evaluation

Formalin-fixed, paraffin-embedded and hematoxylin-eosin (H&E)-stained tumor samples were examined microscopically. Capsule integrity was variable due to the friable nature of the tumors, and sampling included at least one section per centimeter of tumor diameter, representative sections of the capsule and adjacent pancreatic parenchyma. Histopathological evaluation included assessment of mitotic activity, necrosis, lymphovascular invasion (LVI), perineural invasion (PNI), capsule and parenchymal infiltration, and cytological atypia.

### Immunohistochemistry

Tumor-containing paraffin blocks from all patients collected from both centers were processed at a single laboratory for immunohistochemical analysis. Evaluations were performed using standard external control tissues to ensure staining quality and consistency.

Immunohistochemical staining for anti- BAP1 (Clone C-4, 1:100 dilution, Santa Cruz Biotechnology), anti- β-catenin (clone 14, prediluted; Dako, Cell Marque), CD10 (clone SP67, prediluted; Roche), PR (clone 1E2, prediluted; Roche), AR (clone SP107, prediluted; Cell Marque), Chromogranin (clone LKH210, prediluted; Roche), Synaptophysin (clone 27G12, prediluted; Cell Marque), and Ki-67 (clone SP6, predilutated; Cell Marque) were performed on 5-µm-thick sections taken on positively charged slides, using an automated immunohistochemical stainer according to the manufacturer’s guidelines (streptavidin-peroxidase protocol, BenchMark; Ventana, PA). The intensity and extent of staining were evaluated semiquantitatively by two pathologists (EC. and NCD.). For BAP1 immunohistochemistry, staining intensity (0 = none; 1 = weak; 2 = moderate; 3 = strong) and staining extent (0 = 0%; 1 = 1–25%; 2 = 26%–50%; 3 = 51–75%; 4 = 76%–100%) were scored, and the two scores were multiplied to obtain a final score. BAP1 expression was considered negative if the final score was ≤3 and positive if >3. CD10 and synaptophysin were considered positive when ≥1% of tumor cells showed immunoreactivity. β-catenin was considered positive when nuclear and/or cytoplasmic staining was present. Chromogranin expression was considered positive if >1% of tumor cells showed cytoplasmic staining. Ki-67 index was calculated as the percentage of positive tumor cell nuclei.

All histopathological and immunohistochemical evaluations were independently reviewed by two experienced pathologists with subspecialty expertise in surgical pathology, and discrepancies were resolved by consensus.

### Next-generation sequencing (NGS)

NGS was performed on formalin-fixed, paraffin-embedded (FFPE) samples from metastatic and locally aggressive cases (n = 5) using a custom 85-gene panel including *CTNNB1*, *BAP1*, and *AR*. The panel covered the exonic regions and exon–intron junctions of 85 genes. The workflow comprised deoxyribonucleic acid (DNA) extraction, library preparation, sequencing, and bioinformatics analysis. DNA was extracted using the QIAamp DNA FFPE Advanced Uracil-N-glycosylase (UNG) Kit (Qiagen), and concentrations were measured with the Qubit™ dsDNA HS Kit (Thermo). Libraries were prepared with the QIAseq Custom Panel (Qiagen), barcoded, amplified, and purified. Prior to sequencing, library quality was assessed using the QIAxcel Advanced automated electrophoresis system, and libraries were diluted to a final loading concentration of 1 nM. Sequencing was carried out on the AVITI System (Element Biosciences). Secondary analysis and clinical interpretation were performed using Qiagen Clinical Insight–Analyse Universal and Interpret tools.

Microsatellite instability (MSI) status was assessed using the QIAGEN CLC Genomic Workbench software. In addition to the standard loci (BAT40, MONO-27, BAT26, NR24, BAT25, NR22, HSP110-T17, NR21, BAT34C4), the workflow incorporated a broader panel covering 190 loci to ensure comprehensive evaluation.

### Statistical analysis

Categorical variables were assessed using the chi-square test, and nonparametric comparisons between continuous variables and categorical groups were performed using the Mann–Whitney U test in SPSS (version 31.0), where applicable. Survival analyses were generally descriptive due to the low number of events, and exploratory analyses using Firth’s penalized Cox regression were performed in selected instances to mitigate small-sample bias. p values ≤0.05 were considered statistically significant.

### Ethics approval

This retrospective study was approved by the Non-Interventional Clinical Research Ethics Committee of Pamukkale University (Protocol No. 193152). The study was conducted in accordance with the principles of the Declaration of Helsinki, and all data collection and analyses were performed using anonymized information to ensure the protection of patient privacy.

## Results

The demographic and clinical profile of our cohort, characterized by a wide age range (8–71 years), female predominance, and a mean tumor size of 7.2 cm, is summarized in Table 1.

**TABLE 1:** Clinical characteristics and outcomes of patients with SPN (n = 62).

Characteristic	Value
Age (years)	
Mean ± SD	31.3 ± 16.8
Median (IQR)	25.5 (16.0–46.3)
Range	8–71
Sex	
Female	54 (87.1%)
Male	8 (12.9%)
Tumor localization	
Body/tail of pancreas	40 (65.6%)
Head of pancreas	21 (34.4%)
Tumor size (cm)	
Mean ± SD	7.2 ± 4.7
Median (IQR)	6.3 (3.5–9.0)
Range	1.5–22.0
Resection margin	
R0 (intact)	46 (86.8%)
R1 (positive)	7 (13.2%)
Surgical procedure	
Surgical resections^a^	47 (75.8%)
Tumor enucleation	13 (21.0%)
Biopsy/cytology only	2 (3.2%)
Follow-up (months)	Mean 97.2; IQR 105.0
Overall survival	95%
Metastatic disease	4/62 (6.5%)
Locally aggressive disease	4/62 (6.5%)

Abbreviations: IQR, interquartile range; SD, standard deviation; SPN, solid pseudopapillary neoplasm.

^a^ Surgical resection is one of the following: distal pancreatectomy/Whipple procedure/total pancreatectomy/ medial pancreatectomy.

**FIGURE 1:**
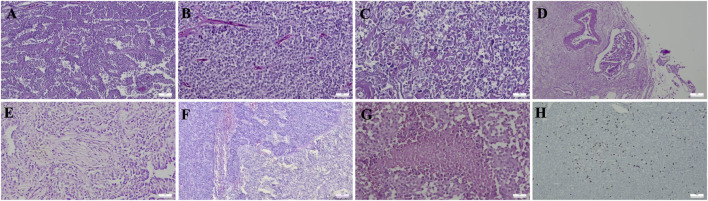
Representative histopathological features of pancreatic solid pseudopapillary neoplasm (SPN). The tumor shows a characteristic admixture of solid and pseudopapillary areas composed of uniform neoplastic cells arranged around delicate fibrovascular cores **(A,B)** (H&E, ×100). Marked cytological atypia is observed in tumor cells **(C)** (H&E, ×200). Lymphovascular invasion **(D)** (H&E, ×100) and perineural invasion **(E)** (H&E, ×200) are illustrated. Pancreatic parenchymal invasion **(F)** (H&E, ×100) and a focus of tumor necrosis **(G)** (H&E, ×200) are shown. An SPN case with a high Ki-67 proliferation index is demonstrated **(H)** (Ki-67 immunohistochemistry, ×100).

Histopathological evaluation was available for most cases and demonstrated LVI in 1 of 59 cases (1.7%) and PNI in 7 of 59 cases (11.9%). Capsular and/or parenchymal invasion was identified in 28 of 59 cases (47.5%). The median Ki-67 index was 5% (range, 1%–15%) in metastatic or locally aggressive SPNs and 2% (range, 0.5%–8%) in other cases. High mitotic activity (>5 mitoses/10 HPF) was observed in 2 of 8 metastatic or locally aggressive cases (25%) and in 1 of 50 other cases (2.0%). The distribution of these parameters according to clinical behavior is summarized in Table 2. Representative histopathological features, including LVI, PNI, and necrosis, were shown in Figure 1.

**TABLE 2:** Clinicopathological features of SPNs according to clinical behavior.

Clinicopathological feature	Metastatic/locally aggressive SPNs (n = 8)	Other SPNs (n = 54)
Tumor size, cm, mean ± SD	7.16 ± 5.93	7.18 ± 4.59
Ki-67 index, %, median (range)	5 (1–15)	2 (0.5–8)
High mitotic activity (>5 mitoses/10 HPF)	2/8 (25%)	1/50 (2.0%)
Necrosis	3/8 (37.5%)	21/50 (42.0%)
LVI	1/8 (12.5%)	0/51 (0%)
PNI	0/8 (0%)	7/51 (13.7%)
Capsular/parenchymal invasion	6/8 (75.0%)	22/51 (43.1%)
Cytological atypia	1/8 (12.5%)	6/52 (11.5%)

Number of evaluable cases varies due to tissue availability.

Abbreviations: HPF, high-power field; LVI, lymphovascular invasion; PNI, perineural invasion; SPN, solid pseudopapillary neoplasm.

Immunohistochemically, all tumors showed nuclear β-catenin expression (100%), most frequently with additional cytoplasmic staining (63.9%). CD10 (95%), PR (84.7%), and AR (82.5%) were consistently expressed, whereas loss of BAP1 was identified in 22.8% of evaluable cases. Synaptophysin (46.8%) and chromogranin (11.3%) were generally only focally positive (Table 3). PR expression was more frequently observed in AR-positive cases (43/47, 91.5%) compared with AR-negative cases (6/10, 60%) (p = 0.025). Chromogranin expression was observed in cases showing synaptophysin expression (85.7%). No significant associations were identified between BAP1, PR, or AR immunohistochemical expression and clinical or histopathological parameters.

**TABLE 3:** Immunohistochemical features of SPN cases (n = 62).

Marker	n (%)	Pattern
β-catenin	61/61 (100%)	Nuclear ± cytoplasmic
​	Nuclear only: 22/61 (36.1%)	​
​	Nuclear + cytoplasmic: 39/61 (63.9%)	​
CD10	57/60 (95.0%)	Membranous/cytoplasmic
BAP1 loss	13/57 (22.8%)	Nuclear loss
PR	50/59 (84.7%)	Nuclear
AR	47/57 (82.5%)	Nuclear
Synaptophysin	29/62 (46.8%)	Cytoplasmic
Chromogranin	7/62 (11.3%)	Cytoplasmic

Number of evaluable cases varies due to tissue availability.

Abbreviations: AR, androgen receptor; BAP1, BRCA1-associated protein 1; PR, progesterone receptor; SPN, solid pseudopapillary neoplasm.

Targeted NGS was successfully performed on primary tumor tissue from five cases with metastatic (n = 2) or locally invasive (n = 3) SPNs. *CTNNB1* exon 3 mutations were identified in all analyzed cases, consistent with the established molecular profile of SPN. Additional molecular alterations were observed in a subset of cases (Table 4). All analyzed cases were microsatellite stable. NGS analysis could not be performed in two metastatic cases (n = 2/4) and one locally invasive case (n = 1/4) due to insufficient tumor material.

**TABLE 4:** Targeted NGS findings in selected metastatic and locally invasive SPNs (n = 5) in our series.

Feature	Findings
*CTNNB1* exon 3 mutations (VAF%)	p.D32N (21%), p.D32N (23%), p.G34R (45%), p.S45P (91%), p.T41I (20%)
Additional molecular alterations (VAF%)	***AR*** p.V731M (7.9%), ***BAP1*** p.R213H (6.7%), ***CDKN2A*** exon 1–2 del, ***ERBB3*** p.E903D (47%), ***FBXW7*** (10%), ***MLH1*** p.R265C (14%), ***NF1*** del, ***SETD2*** (10%), ***TP53*** del (24%)
MSI status	All cases MSS

Abbreviations: del, deletion; MSI, microsatellite instability; MSS, microsatellite stable; p., protein-level change according to Human Genome Variation Society (HGVS) nomenclature; SPN, solid pseudopapillary neoplasm; VAF, variant allele frequency.

The overall survival rate was 95% (57/60) at a mean follow-up of 97.2 months. Aggressive clinical behavior was observed in eight cases (12.9%), including distant metastasis in four patients (6.5%) and locally aggressive disease in four patients (6.5%). Metastatic cases comprised three synchronous metastases involving the lung, liver, or lymph nodes and one metachronous relapse presenting as liver metastasis 6 months after surgery. Locally aggressive disease involved adjacent structures such as the duodenum, ampulla of Vater, portal vein, or splenic vein, and all of these patients remained relapse-free during follow-up. Overall, three patients (5.0%) died during the follow-up period.

## Discussion

In our series, patients exhibited a wide age range with a marked female predominance and relatively large tumors (mean size, 7.2 cm), aligning with the demographic profile described in previous studies [12, 15]. SPN is generally regarded as a neoplasm with low malignant potential and a favorable prognosis, reflected by reported 5-year survival rates of approximately 97% [12]. In our study, overall survival rate was 95%, consistent with previous reports [16, 17]. The overall metastatic rate in our cohort was 6.5% (4/62), which is lower than the 10%–15% range reported in the literature. The low metastatic rate observed in our cohort may be related to the absence of complete 5-year follow-up for all patients.

The terminology used to describe SPNs with progressive behavior remains inconsistent across the literature, with terms such as “malignant,” “high-grade,” or “metastatic” SPN often used interchangeably [18, 19]. In line with the current WHO classification framework and to avoid overinterpretation, we used the descriptive term “SPNs with aggressive clinical features” for cases presenting with metastatic disease or local invasion [20]. Several clinicopathological features have been reported in the literature in association with aggressive clinical behavior in SPN, including large tumor size, local invasion, distant metastasis, and increased proliferative activity [12, 18, 21–23]. In the present series, tumor size did not differ significantly between SPNs with metastatic or locally aggressive features and other cases. Nevertheless, in an exploratory survival analysis, increasing tumor size was associated with less favorable outcomes using Firth’s penalized regression (hazard ratio, 1.31 per cm increase; 95% CI, 1.05–1.62), a finding that requires confirmation in larger, independent cohorts.

The association between histopathological features and clinical behavior in SPN has been extensively studied. The WHO classification recognizes “solid pseudopapillary neoplasm with high-grade carcinoma” as a distinct histologic subtype associated with aggressive clinical behavior and adverse outcomes, representing an uncommon subset of SPNs [20]. In our series, one case met these criteria, showing marked cytologic atypia and a high mitotic index, presenting with metastatic disease at diagnosis and resulting in death 19 months later. Undifferentiated sarcomatoid areas, which have been associated with unfavorable outcomes in previous reports [12], were not observed in our series. Increased mitotic activity, LVI, PNI, and tumor necrosis have been most frequently discussed in the literature in relation to aggressive clinical behavior in SPN [12, 24]. In our series, increased mitotic activity and capsular/parenchymal invasion were more commonly observed in metastatic or locally aggressive cases; however, given the limited number of such cases, no prognostic analyses were performed. PNI has been reported sporadically in SPNs, but available series indicate that it is uncommon and its prognostic significance remains uncertain [18, 25]. Consistent with these observations, PNI was not identified in any metastatic or locally aggressive cases in our series, suggesting that PNI alone is unlikely to be a reliable marker of aggressive clinical behavior in SPN.

Immunohistochemically, PR expression is well established in SPNs and represents a characteristic component of the immunophenotype, in addition to nuclear β-catenin expression [9]. Increased PR expression may be related to hormone-associated signaling and biological activity. AR, together with PR, forms part of the hormone receptor spectrum in SPNs and may be involved in hormone-related signaling pathways interacting with Wnt/β-catenin signaling [26]. In our cohort, PR expression was more frequently observed in AR-positive cases. BAP1 loss has been reported in a subset of SPNs, particularly in metastatic cases, although its biological and clinical significance remains incompletely understood [10]. In the present series, BAP1 loss was observed in 22.8% of cases. Ki-67 indices were generally low in our series (median 2%), consistent with previous reports. While higher Ki-67 thresholds have been discussed in the literature in relation to aggressive behavior, no consensus cut-off has been established [18, 27]. In our study, Ki-67 index values tended to be higher in metastatic or locally aggressive cases, although the small number of events precluded any definitive prognostic assessment.

*CTNNB1* exon 3 mutations are the key driver of SPN [5, 6, 10]. Tipmanee et al. reported that exon 3 mutations of the *CTNNB1* gene, particularly those involving the D32 codon, result in a charge alteration within the phosphorylation motif, leading to loss of affinity for the axin–Glycogen synthase kinase-3 beta complex in SPN [28]. Fleming et al. further observed that D32N/Y/H variants were found exclusively in metastatic SPNs (n = 11), whereas S37F/C mutations predominated in primary tumors (n = 17) [8]. These substitutions affect distinct regulatory motifs within exon 3, with D32 located in the β-TrCP recognition site, potentially stabilizing β-catenin by preventing its ubiquitin-mediated degradation. In the present series, the D32N variant was identified in two cases. The clinical relevance of specific exon 3 substitutions requires further investigation in larger cohorts. The most frequently reported molecular alterations specifically identified in solid pseudopapillary neoplasms with aggressive clinical features are summarized in Table 5, whereas a broader overview of gene-level molecular alterations and associated pathways reported across SPNs in published NGS studies is provided as Supplementary Table S1 due to space limitations.

**TABLE 5:** The most frequently reported molecular alterations in SPNs with aggressive clinical features.

Gene	Alteration pattern	Functional context	Key references
*CTNNB1*	Exon 3 activating hotspot mutations	WNT/β-catenin pathway activation	[8, 10, 29]
*KDM6A*	Inactivating variants or loss of expression	Epigenetic regulation	[10]
*BAP1*	Loss-of-function variants or protein loss	Chromatin remodeling	[8, 10]
*TET1*	Inactivating variants	DNA methylation/epigenetic control	[10]
*TP53*	Pathogenic variants	Cell cycle/DNA damage response	[29]

Abbreviations: DNA, deoxyribonucleic acid; WNT, Wingless/Integrated signaling pathway.

In this study, the additional molecular alterations detected were consistent with previous reports describing rare secondary events in SPNs. Classical SPNs typically lack the hallmark mutations of pancreatic ductal adenocarcinoma, such as *KRAS* and *SMAD4* [30], although occasional alterations involving *TP53* and *CDKN2A* have been reported in cases with aggressive clinical features [19]. In our series, no *KRAS* or *SMAD4* mutations were identified. *BAP1* inactivation has also been reported in association with aggressive or metastatic SPNs [10]. Other reported co-alterations involve genes related to cell cycle regulation and DNA repair pathways, including *NF1*, *FBXW7*, *MLH1*, and *BRCA2*, generally occurring at low frequency [29, 31]. To our knowledge, the *SETD2* splice-site variant, *ERBB3* p.E903D, and *AR* p.V731M identified in this cohort have not been previously described in published SPN series or case reports, including genomic analyses of primary and metastatic SPNs (Table 4) [6, 8, 10, 29].

Surgery remains the cornerstone of SPN management, while no standard systemic therapy has been established for unresectable or clinically aggressive cases. Previous case reports and sequencing-based studies have described various molecular alterations in SPN and have discussed potential pathway-level vulnerabilities; however, these observations remain exploratory [32–34]. In the present cohort, *CTNNB1* exon 3 mutations were consistently identified, accompanied by additional low-frequency alterations involving *BAP1*, *CDKN2A*, *NF1*, and *TP53* (Table 4). These findings reflect molecular heterogeneity rather than the presence of defined therapeutic targets.

Regarding immunotherapy, SPNs are typically microsatellite stable with low tumor mutational burden, including in our series and previously reported cohorts [10, 30]. The clinical role of immune checkpoint inhibition in SPN appears limited and remains unclear. Overall, the limited and heterogeneous molecular data underscore the need for larger, collaborative studies to better define the biological and clinical relevance of these alterations.

This study has several limitations, including its retrospective design, the relatively small number of cases with aggressive clinical features, and the limited availability of tissue for molecular testing, which restricted NGS analysis to a subset of patients. Although follow-up duration was long, the low number of adverse outcome events limited the ability to explore robust associations between histopathological parameters and clinical outcomes. These limitations warrant careful contextualization of the findings and underscore the need for validation in larger, multi-institutional cohorts.

## Conclusion

This study presents a clinicopathological, immunohistochemical, and molecular evaluation of SPNs, with particular focus on cases showing locally aggressive or metastatic behavior. The findings demonstrate the wide histopathological spectrum of these tumors and confirm the central role of *CTNNB1* exon 3 mutations as a common molecular feature. In addition, a range of other molecular alterations was identified, although these occurred infrequently and without a consistent pattern.

Rather than defining a specific molecular marker associated with aggressive behavior, the results point to considerable biological variability among advanced cases. Taken together, the data suggest that aggressive behavior in solid pseudopapillary neoplasms cannot be explained by a single additional genetic or protein-level alteration. Further studies including larger, multi-institutional cohorts will be necessary to better understand the clinical relevance of these heterogeneous findings.

## Data Availability

The datasets presented in this study can be found in online repositories. The names of the repository/repositories and accession number(s) can be found in the article/Supplementary Material.

## Supplementary material

SUPPLEMENTARY TABLE S1 Reported gene-level molecular alterations and associated pathways in solid pseudopapillary neoplasms with aggressive clinical features based on published NGS studies.
